# Enhancing interpreting performance, engagement, and self-regulated learning through peer assessment: an attachment theory perspective

**DOI:** 10.3389/fpsyg.2025.1604825

**Published:** 2025-12-05

**Authors:** Chunwen Yang, Shuai Hou, Jing Chen

**Affiliations:** 1 School of Advanced Translation and Interpretation, Dalian University of Foreign Languages, Dalian, China; 2 School of English Studies, Dalian University of Foreign Languages, Dalian, China

**Keywords:** attachment theory, interpreter training, learning engagement, peer assessment, self-regulated learning (SRL)

## Abstract

This study addresses the challenge of limited personalized feedback in interpreter training, which often hinders students’ engagement, self-regulation, and performance development. Guided by Attachment Theory, it explores how peer assessment can serve as a secure relational scaffold that enhances learning outcomes. Employing a quasi-experimental design with 30 undergraduate interpreting students divided into experimental and control groups, the study combined pre- and post-tests with surveys and thematic analysis to evaluate interpreting performance, engagement, and self-regulated learning (SRL). Quantitative results showed significant improvement in interpreting performance, engagement, and SRL in the peer-assessment group, while qualitative findings revealed improved confidence, reflective thinking, goal-setting, and peer support. Peer assessment fostered emotional safety, motivation, and strategic learning behaviors that enhanced both engagement and SRL. These findings imply that attachment-informed peer feedback can strengthen interpreters’ cognitive, affective, and behavioral learning dimensions, promoting sustainable skill development and learner autonomy. Overall, the study underscores the pedagogical value of designing peer assessment practices that balance evaluative rigor with psychological safety in interpreter training.

## 1 Introduction

Student engagement is a multidimensional construct encompassing behavioral, emotional, and cognitive dimensions, all of which contribute to meaningful learning outcomes (Gao and Fan, 2024; Liu and Aryadoust, 2024; Wong and Liem, 2022; Xue and Liu, 2024). Students with high level of engagement invest sustained effort, display curiosity, and demonstrate persistence when facing challenges, which in turn enhances academic achievement and skill acquisition. In higher education, engagement is closely linked to students’ willingness to interact with feedback and take active roles in their own learning processes (Zhang and Hyland, 2022). Perceived teacher support can further reinforce engagement by fostering motivation, attentiveness, and resilience (Tao et al., 2022). Alongside engagement, self-regulated learning (SRL) refers to learners’ ability to set goals, select strategies, monitor progress, and adapt approaches to optimize performance (Li and Lajoie, 2022). Recent research demonstrates that SRL improves learning outcomes across disciplines (Xu et al., 2023) and supports the development of complex skills, such as language proficiency (Öztürk and Çakıroğlu, 2021; Yang et al., 2024). Thus, engagement and SRL form a foundation for effective and sustainable learning, enabling students to accomplish greater academic growth.

One prevailing pedagogical solution to enhancing both engagement and SRL is peer assessment, where learners evaluate and provide feedback on each other’s work using shared criteria (Holewik, 2020; Iglesias Pérez et al., 2020; Lee, 2016; Li et al., 2023; Wu and Schunn, 2023; Zhao et al., 2023). By engaging in critical reflection, justifying evaluations, and offering constructive suggestions, students activate metacognitive strategies that directly support self-regulation (Ortega-Ruipérez and Correa-Gorospe, 2024). Peer feedback activities including those supported by educational technology can promote motivation, collaborative learning, and the transfer of strategies across tasks (Tran and Qing, 2024; Yin et al., 2022). Moreover, according to Attachment Theory, learning is strongly influenced by the presence of secure emotional bonds, which provide a foundation of trust, empathy, and consistent support (Bretherton, 1985). It offers an important lens for understanding the interpersonal dynamics of peer assessment, as secure and supportive peer relationships foster psychological safety, openness to criticism, and a willingness to experiment with new strategies (London et al., 2023; Toffoli, 2015). When students feel emotionally secure in their peer networks, they are more receptive to feedback, more likely to engage actively in dialogue about performance, and more confident in applying improvements. This relational dimension of peer assessment thus extends its value beyond skill development to include affective and motivational benefits that underpin deeper learning.

Interpreting is a cognitively demanding and performance-driven practice requiring the rapid processing, comprehension, and reformulation of spoken messages under strict time constraints (Gile and Lei, 2020; Mei and Yang, 2025; Zou et al., 2025). Feedback therefore plays a critical role in improving interpreting performance, guiding learners to identify errors, refine techniques, and develop professional standards (Du and Salaets, 2025; Lee J., 2018; Lee S. B., 2018; Yang, 2025). However, in many interpreter training contexts, due to the large number of students, interpreter trainers cannot always provide personalized, immediate feedback to each student and in most cases, students have to record their voice, recall back their performance, and give feedback by their own. This limitation can leave gaps in learners’ understanding of their performance and hinder timely skill refinement. In this regard, peer assessment offers a practical and pedagogically sound solution to this challenge, enabling students to receive more frequent and diversified feedback while simultaneously cultivating their evaluative judgment and reflective abilities. Through structured peer review, students interpreters can deepen their awareness of performance criteria, learn from the varied strategies of their peers, and sustain engagement in continuous improvement.

Thus, given the roles of engagement, SRL, and supportive peer relationships in skill-based learning, the present study aims to examine the impact of peer assessment on student interpreters’ performance, engagement, and SRL, using Attachment Theory as the guiding framework. By exploring how structured peer feedback influences both cognitive and affective dimensions of interpreter training, the study addresses a gap in the current literature where the relational and motivational aspects of peer assessment in interpreting training remain underexplored. The findings hence have the potential to inform interpreter trainers on how to integrate peer assessment more effectively into curricula, balancing the need for professional standards with the cultivation of supportive learning communities. Ultimately, this research seeks to contribute to the development of pedagogical strategies that improve interpreting skills and foster lifelong learning dispositions essential to the interpreting as a profession.

## 2 Literature review

### 2.1 Peer assessment

Peer assessment has been widely recognized as a pedagogical strategy for promoting student learning by fostering active engagement, metacognitive awareness, and self-regulation (Massey and Brändli, 2016; Ortega-Ruipérez and Correa-Gorospe, 2024; Chen and Han, 2025). While traditional teacher-led feedback often positions learners as passive recipients of information, peer feedback on the contrary, positions students as both knowledge producers and evaluators, enhancing their capacity to internalize quality criteria and apply them in their own work (Er et al., 2020; Cui et al., 2021). Previous studies consistently report that structured and trained peer feedback can yield gains in performance, self-efficacy, and autonomous motivation comparable to or exceeding teacher feedback (Cui et al., 2021; Cheng et al., 2023). Additionally, Cao et al. (2022) reveal that online peer feedback through technology-mediated platforms contributes to iterative revisions, rubric-guided evaluation, and timely feedforward. However, learning effectiveness depends on systematic curriculum design and with clear guidance, anonymity protocols, and structured criteria (Er et al., 2020; Ortega-Ruipérez and Correa-Gorospe, 2024).

Moreover, recent studies also reveal the affective and social dimensions of peer assessment, highlighting the role of trust, psychological safety, and emotional engagement in shaping learning outcomes (Jongsma et al., 2024; Xu and Zhang, 2025). While anonymity can protect students from peer pressure, dialogic interaction fosters richer feedback exchanges and deeper reflection, suggesting a need to balance safety with interactivity (Jongsma et al., 2024). In addition, mobile-assisted and computer-mediated environments have further diversified peer assessment practices, enabling learners to provide oral or written feedback in both first and target languages, which can enhance speaking performance, reduce anxiety, and build willingness to communicate (WTC) (Ebadijalal and Yousofi, 2021). From a sociocultural perspective, peer feedback engages learners in dynamic affective, cognitive, and behavioral processes, where emotions can catalyze or constrain engagement (Xu and Zhang, 2025). However, while these studies affirm the academic and motivational benefits of peer assessment, uneven feedback quality, variable student commitment, and emotional vulnerabilities remain central issues in evaluating the learning effectiveness of peer assessment.

### 2.2 Engagement

Student engagement plays a crucial role in students’ learning outcomes in language learning (Yu et al., 2025). It has been widely recognized as a multidimensional construct encompassing behavioral, cognitive, emotional, and social facets (Sun and Zhang, 2024). Studies have shown that engagement is shaped by internal motivational factors and external support systems. For instance, Luan et al. (2020) demonstrated that perceived teacher and peer support significantly enhance engagement, with behavioral engagement mediating its effects on cognitive, emotional, and social engagement. Similarly, Liu and Feng (2023) and Guo et al. (2025) found that both academic and emotional teacher support predict learning engagement, though the mechanisms differ. Academic support operates through self-efficacy and achievement goals, while emotional support functions primarily through mastery goals. In addition, the quality of teacher–student interaction also matters. For example, Dewaele and Li (2021) revealed that teachers’ enthusiasm influences students’ social-behavioral engagement indirectly by increasing enjoyment and reducing boredom. In blended learning environments, Huang et al. (2022) noted that activity type and teacher role affect emotional and cognitive engagement differently, showing that engagement is context-sensitive and mediated by instructional design.

Beyond the influence of teaching and context, learner engagement also depends on how students interact with feedback and regulate their learning processes. For instance, Liu and Feng (2023) emphasized that engagement is indispensable for the effectiveness of corrective feedback, advocating for explicit strategies to help learners work with feedback. Wang et al. (2025) expanded on this by detailing cognitive, behavioral, and affective engagement with teacher oral feedback, noting that while students generally respond positively, engagement levels vary depending on self-regulatory capacity. Moreover, engagement is also closely linked to psychological well-being. For example, Cong et al. (2024) showed that higher engagement predicts lower burnout among EFL students, partly by enhancing academic self-efficacy. Furthermore, Sun and Zhang (2024) observed that while multiple engagement dimensions correlate with outcomes, actual behavioral engagement such as task completion, was the strongest predictor of performance, signaling the importance of measurable, observable actions. Thus, these findings suggest that fostering engagement requires creating supportive, emotionally positive learning environments and equipping students with the skills and dispositions to actively process feedback, sustain motivation, and manage their own learning.

### 2.3 Self-regulated learning

Self-regulated learning (SRL) has been identified as a decisive factor in fostering learner autonomy, sustained motivation, and improved academic performance in various EFL contexts. Prior studies show that SRL strategies, ranging from goal setting and self-monitoring to feedback handling and motivational regulation, positively influence skill development across writing, speaking, and integrated language tasks (Sun and Wang, 2020; Öztürk and Çakıroğlu, 2021; Bai and Wang, 2023). SRL effectiveness, however, often hinges on learners’ self-efficacy and their ability to strategically deploy cognitive and metacognitive resources. For example, Xu et al. (2024) found that SRL strategies in smart classrooms do not directly predict engagement but operate through the mediating role of self-efficacy, suggesting that confidence in task completion is a prerequisite for sustained strategy use. Therefore, teacher- and peer-mediated interventions have emerged as effective scaffolds for SRL development. For instance, Yang et al. (2022) and Zimmerman (2002) showed that SRL-based feedback interventions significantly improved students’ goal-oriented monitoring, idea planning, and emotional control, while Kumar et al. (2023) demonstrated that both self- and peer-assessment activities significantly enhanced SRL, critical thinking, and problem-solving skills.

Additionally, recent research has also examined how different feedback modalities and learning designs shape SRL processes in different ways. Tian et al. (2022) and Tran and Qing (2024) found that feedback source influences the type of SRL strategies activated as automated feedback tends to elicit more cognitive strategies, while teacher feedback prompts greater motivational regulation, and peer feedback fosters collaborative reflection and help-seeking. Similarly, online and blended learning contexts present both challenges and opportunities for SRL. For example, Rasheed et al. (2021) demonstrated that scaffolding peer-learning SRL strategies in the online component of blended learning can mitigate social loafing and significantly improve performance. Intervention studies further reveal that reflective and portfolio-based approaches (Zhang and Chieh, 2025) and online peer feedback (Öztürk et al., 2025) can cultivate metacognitive awareness, responsibility for learning, and strategic planning. Yan (2019) adds that self-assessment plays a pivotal role across SRL phases, with performance-phase monitoring and feedback-seeking particularly predictive of achievement. These studies affirm that SRL is a dynamic process shaped by motivational beliefs, contextual affordances, and the quality of learner–feedback interaction.

### 2.4 Attachment theory

Rooted in Bowlby’s ethological account, attachment theory explains how proximity-seeking to reliable others organizes behavior under stress and scaffolds exploration (Bowlby, 1979). Ainsworth’s refinements and Bretherton’s synthesis foreground internal working models such as expectancies about self and others that regulate attention, affect, and help-seeking in learning contexts (Bretherton, 1985). In adulthood, these models manifest as secure, anxious, or avoidant strategies of affect regulation with clear cognitive consequences that security promotes openness and flexible problem solving, whereas insecurity biases attention, narrows strategy use, and amplifies defensive responding (Mikulincer et al., 2003). Evidence from clinical and community settings shows that secure attachment relates to better emotion regulation and engagement, and that systems which cultivate predictable, empathic relationships support adaptive functioning (Goodwin, 2003; Shoshani et al., 2014). Theoretically, attachment dovetails with motivation frameworks that under stress, secure learners tend to pursue learning goals and constructive strategies, while insecure learners gravitate toward self-validation and defensive coping (Rusk and Rothbaum, 2010). Despite this strong conceptual match with classroom processes such as feedback uptake, risk-taking, and self-regulation, language education has only sporadically leveraged attachment as an explanatory lens, with notable calls to relate learner autonomy and out-of-class learning to attachment-derived differences in help-seeking and exploration (Toffoli, 2015). In short, the field recognizes that emotion and relationship climates shape engagement, yet attachment-informed designs remain under-theorized and under-tested in L2 settings.

Peer assessment is a promising lens for attachment-informed pedagogy. Dialogic peer feedback can be structured to move from socially shared regulation to co-regulation and, ultimately, self-regulation (Domínguez Araújo, 2019). However, its benefits depend on psychological safety, trust, and constructive relational norms (Er et al., 2020; London et al., 2023). Studies in L2 writing and speaking show that well-trained peer feedback can match teacher feedback on competence gains, bolster autonomous motivation, reduce anxiety and deepen behavioral, cognitive, and affective engagement (Cui et al., 2021; Ebadijalal and Yousofi, 2021; Cao et al., 2022; Cheng et al., 2023; Xu and Zhang, 2025). Interpreter training adds two critical divergence that students value teacher authority yet seek emotional support, and peer assessment quality varies with rater expertise and task features, therefore call for explicit training and dialogic safeguards (Lee J., 2018; Lee S. B., 2018; Fowler, 2007; Han, 2018a,b; Han and Zhao, 2020; Jongsma et al., 2024). Thus, attachment-sensitive design with anonymity where needed, clear rubrics, iterative dialogue, and feedback literacy can convert peer work into a secure relational scaffold that enhances WTC, emotion regulation, and SRL in language learning. The critical research direction is to operationalize attachment constructs within peer-feedback ecologies in L2 and interpreting classrooms, exploring how security-promoting features such as predictable routines, empathetic discourse moves mediate learning gains and reduce the variance in rater accuracy and feedback uptake.

### 2.5 Gaps

Previous research demonstrates that peer assessment and SRL each play important roles in enhancing EFL learners’ performance, motivation, and autonomy, yet studies have largely examined these constructs in isolation or in loosely connected pairs. Peer feedback research has advanced frameworks for dialogic exchange (Er et al., 2020) and documented its cognitive, affective, and behavioral benefits (Cui et al., 2021; Xu and Zhang, 2025), yet little is known about how such feedback processes directly interact with learners’ engagement patterns and SRL strategies. Similarly, engagement studies highlight the influence of social support, teacher enthusiasm, and activity design on behavioral, cognitive, emotional, and social dimensions (Luan et al., 2020; Dewaele and Li, 2021), but they rarely address how engagement mediates or is mediated by peer-feedback activities in professional skill training such as interpreting, which involves high cognitive load, real-time performance, and interpersonal skills. In addition, research on SRL confirms that strategy use and self-efficacy are crucial for language learning success (Sun and Wang, 2020; Zhang and Chieh, 2025), but few studies have investigated how peer feedback can serve as a catalyst for SRL development in interpreting contexts, where rapid self-monitoring and adaptive problem-solving are essential.

Moreover, the integration of these three domains remains underexplored in terms of their dynamic, reciprocal relationships and their combined impact on learning outcomes in interpreting training. Existing studies tend to focus on general EFL or writing skills without systematically addressing the unique demands of interpreting, such as sustained attention, split processing, and management of performance anxiety. The affective dimension, while recognized as influential in both engagement (Dewaele and Li, 2021) and feedback uptake (Xu and Zhang, 2025), has not been deeply examined through the lens of Attachment Theory, which could explain how learners’ perceived relational security with peers and instructors influences their willingness to give, receive, and act upon feedback in high-pressure interpreting tasks. Additionally, much of the current evidence is context-specific, frequently limited to short-term interventions, single skill areas, or particular technologies, leaving open questions about the transferability of findings to interpreting classrooms and their applicability across cultural settings. This gap points to the need for research that examines how peer feedback, guided by Attachment Theory, can be designed to foster sustained engagement and SRL in student interpreters, while accounting for the interplay of cognitive, behavioral, and affective processes in this specialized and performance-intensive learning environment.

### 2.6 The present study

Building on the identified gaps in the literature, the present study investigates the role of peer assessment in the context of interpreter training. While prior research has examined peer feedback, engagement, and SRL in general language learning contexts, few studies have integrated these constructs within interpreting training or examined them through the lens of Attachment Theory. Attachment Theory provides a useful framework for understanding how interpersonal dynamics, trust, and perceived security in peer interactions may influence learning behaviors and affective responses. By situating peer assessment in an interpreting classroom, this study aims to explore its associations with interpreting performance, learning engagement, and SRL, offering insights into how feedback processes can be optimized for specialized, high-stakes language tasks. Therefore, the present study aims to answer the following research questions:

*RQ 1*: Is peer assessment associated with improved interpreting performance among student interpreters?

*RQ 2*: Is peer assessment associated with improved learning engagement among student interpreters?

*RQ 3*: Is peer assessment associated with improved self-regulated learning among student interpreters?

## 3 Methodology

### 3.1 Research design

The purpose of this study is to explore the impact of peer assessment on interpreter students’ interpreting performance, engagement and SRL, underpinned by Attachment Theory. To achieve this objective, a quasi-experimental design was adopted, involving two groups: an experimental group and a control group, each consisting of 15 undergraduate students enrolled in a Reading and Interpreting class. The experimental group participated in structured peer assessment activities throughout the class, while the control group received only traditional teacher-led feedback. To evaluate students’ interpreting performance, both groups completed a pre-test and post-test assessed by professional raters using a standardized rubric. In addition, students in the experimental group completed a post-intervention survey that included both closed-ended and open-ended survey questions (Zarei Hajiabadi et al., 2022). This mixed-methods design allowed for a comprehensive comparison of learning outcomes between the two groups, while also capturing students’ subjective experiences with peer assessment in relation to engagement and SRL.

### 3.2 Participants

To achieve the research objective of examining the associations between peer assessment, interpreting performance, learning engagement, and SRL in an interpreting training classroom, the present study targeted second-year undergraduate students majoring in translation at Dalian University of Foreign Languages. Due to the limited class size of the targeted interpreting course in the study semester, only 30 students (7 male, 23 female) were recruited. While the sample size was constrained by course enrollment, prior research suggests that meaningful thematic saturation can often be achieved with relatively small participant numbers. For example, Guest et al. (2006) observed that core themes typically emerge within the first 12 interviews, with additional data providing refinement rather than fundamentally changing the findings. This supports for the adequacy of the present sample in generating valid and meaningful qualitative results. However, the authors hope that, with the expansion of enrollment in translation and interpreting programs, future research can be conducted with a larger sample size to enhance the generalizability of findings.

All participants were native Chinese speakers with good English proficiency and a foundational understanding of interpreting, acquired through prior coursework and training. Participants were assigned to two groups (see Table 1), an experimental group (*n* = 15) and a control group (*n* = 15) using systematic sampling based on student ID to ensure balanced and objective group allocation (Ballance, 2024; Mahama et al., 2022; Wilhelm et al., 2017). To facilitate ongoing collaboration and minimize disruption from absences or withdrawals, the experimental group was further divided into five smaller peer assessment sub-groups, each comprising three members. The control group consisted of 15 students who did not participate in peer assessment activities.

**Table 1:** Participant group and sub-group allocation.

Group	Sub-group	Participant
Experiment Group	Group 1	I01, I11, I21
	Group 2	I03, I13, I23
	Group 3	I05, I15, I25
	Group 4	I07, I17, I27
	Group 5	I09, I19, I29
Control Group	Group 6	I02, I04, I06, I08, I10, I12, I14, I16, I18, I20, I22, I24, I26, I28, and I30

### 3.3 Experiment

#### 3.3.1 Pre-test

Before the intervention, a pre-test was conducted to evaluate each student’s interpreting performance and establish a baseline for comparison. All 30 students were assessed by two experienced interpreting trainers using a standardized rubric adopted from Lee (2015), with a total score of 100 points. The rubric consisted of three key dimensions: Content, Form, and Delivery, each comprising seven marking criteria. The Content dimension focused on accuracy, completeness, and coherence; the Form dimension assessed grammatical correctness, clarity, and appropriate use of language; and the Delivery dimension evaluated fluency, confidence, and time management. The structured rubric used for both peer and teacher assessment is provided in Appendix I. The final score for each student was calculated by averaging the two interpreter trainers’ ratings. As suggested by Shabara et al. (2024), a two-way random effects model with absolute agreement was used to assess inter-rater reliability. The results showed moderate-to-good consistency between raters, ICC(2,k) = 0.66, 95% CI [0.29, 0.84], *p* = 0.002, thereby confirming acceptable inter-rater reliability.

#### 3.3.2 Intervention

The intervention was conducted during the 2024 fall semester over 19 weeks, with the experimental procedures implemented from Week 5 to Week 13. In the first 4 weeks, all students received instruction on fundamental theories and practical techniques in reading, translation and interpreting, ensuring a common foundational competence before the experimental phase began. From Week 5 onward, both groups completed interpreting activities according to the schedule in Table 2. The interpreting materials were designed to resemble authentic professional scenarios and were adapted from instructor’s prior interpreting experiences. An excerpt of the training materials is provided in Appendix II. Each task type differed in length and complexity: dialogues averaged about 50 words per exchange, mini-talks approximately 100 words, and speeches 150 to 200 words. Most vocabulary items were familiar to students. For specific terms, a 5-min glossary preview was provided before each session. Task difficulty was calibrated to maintain comparable cognitive demand across weeks. All students were allowed to take notes freely during interpreting. Note-taking was treated as an integral part of performance practice and feedback from peers and the instructor occasionally addressed note-taking effectiveness as part of qualitative comments. Each class session lasted about 45 min, including roughly 25 min of interpreting practice and 20 min of assessment.

**Table 2:** Intervention Schedule.

No.	Time	Topic	Activity	Duration
1	Week 5	Tourism	Dialogue Consecutive Interpreting	45 min (≈25 min training practice; ≈20 min assessment)
2	Week 6–7	Education	Mini Talk Consecutive Interpreting	
3	Week 8–9	Culture	Mini Talk Consecutive Interpreting	
4	Week 10–11	Science and Technology	Speech Consecutive Interpreting	
5	Week 12–13	Environmental Protection	Speech Consecutive Interpreting	

All interpreting activities for each week involve 1 English to Chinese session and 1 Chinese to English session prepared for respective topics.

Students in the experimental group engaged in peer assessment immediately after performing their tasks. They used a structured rubric adopted from Lee (2015) and provided written feedforward comments to guide their peers’ future improvement. Prior to the intervention, students received brief training on how to apply the rubric, review sample performances, and conduct calibration and practice rounds to ensure consistency and reliability in peer feedback. The control group completed the same interpreting tasks but received feedback solely from the instructor, who also used the same rubric for evaluation. Due to time limitations, not all control-group students received detailed in-class comments each week. However, the instructor maintained assessment records to ensure that every student was provided with individualized feedback at least once every two class sessions. This design allowed a controlled comparison between peer-assessed and teacher-assessed interpreting practice, examining their respective effects on students’ performance, engagement, and SRL.

#### 3.3.3 Post-test

Following the instructional intervention, a post-test was conducted to measure any changes in interpreting performance. The format, procedure, and scoring rubric were identical to those used in the pre-test to maintain consistency. Each student was evaluated by the same two interpreter trainers. Following the same procedure, inter-rater reliability for the post-test was examined using ICC(2,k), which indicated moderate agreement between raters, ICC(2,k) = 0.56, 95% CI [0.01, 0.80], *p* = 0.002. The final score was again calculated as the average of their ratings. This post-test provided a reliable basis for comparing performance improvement between the experimental and control groups and for assessing the overall effectiveness of the peer assessment intervention.

#### 3.3.4 Post-intervention survey

To explore the students’ perceptions of peer assessment and its impact on their interpreting performance, engagement, and SRL, a post-intervention survey was conducted with all students in the experimental group (*n* = 15). The survey was designed in alignment with attachment theory, emphasizing the emotional and social dimensions of peer interaction in learning. It consisted of both closed-ended and open-ended questions. The closed-ended questions (*n* = 9) focused on students’ emotional support, confidence, participation, reflection, and learning behaviors, comparing their experiences with peer assessment and traditional teacher-led feedback. These questions were designed to capture students’ agreement on the usefulness and emotional impact of peer assessment. The open-ended questions (*n* = 9) invited students to elaborate on their experiences with peer relationships, feedback usefulness, changes in learning strategies, and emotional or motivational differences between peer and teacher feedback. Furthermore, in order to ensure content validity, the survey questions were sent to an expert in the same educational field before distributing to the students.

### 3.4 Data analysis

All quantitative analyses were conducted using SPSS (version 26) and JASP (version 0.95.4, Apple Silicon). SPSS was used to calculate the Intraclass Correlation Coefficient (ICC) to assess inter-rater reliability based on a two-way random effects model with absolute agreement. Independent-samples t-tests were employed to assess group equivalence and potential differences, while the Shapiro–Wilk test was used to verify the normality of score distributions. When assumptions of normality were uncertain, Mann–Whitney U tests were performed as a robustness check. To examine the effects of time (pre-test vs. post-test) and group (experimental vs. control), a 2 × 2 mixed ANOVA was conducted (Ajabshir, 2024; Zou et al., 2025). Descriptive statistics, including frequencies and percentages, were also calculated for the closed-ended survey items to summarize students’ views on how peer assessment contributed to their engagement, emotional support, and SRL.

Open-ended survey responses were examined through thematic analysis following the constant comparative method (Boeije, 2002). Data were manually coded and reviewed to identify recurring ideas, distinctive perspectives, and emerging conceptual patterns. Codes were refined iteratively and organized into overarching themes and subthemes, each illustrated with representative quotes. A conceptual analysis approach (Byrne, 2022) was also applied to interpret the meanings associated with students’ perceptions of peer feedback in relation to performance, engagement, and SRL. The analysis followed a three-phase process of transcription, coding, and thematic interpretation (Braun and Clarke, 2012; Cresswell, 2013). The initial coding was completed by the first author, with verification and refinement by the second and third authors. Any discrepancies were resolved through discussion, ensuring analytical consistency and depth of interpretation.

## 4 Results and findings

### 4.1 Relating to RQ1

#### 4.1.1 Quantitative results

To answer RQ1 (Is peer assessment associated with improved interpreting performance among student interpreters?), independent-samples *t*-tests were carried out to examine potential pre-existing differences between the experimental group and the control group in demographic and baseline performance measures. No significant differences emerged between groups in gender distribution, *t*(28) = −0.42, *p* = 0.68, age, *t*(28) = −0.57, *p* = 0.57, or pre-test interpreting scores, *t*(28) = −0.16, *p* = 0.87. The EG (*M* = 74.67, SD = 2.19) and CG (*M* = 74.80, SD = 2.27) demonstrated comparable interpreting proficiency prior to the intervention, indicating that both groups were equivalent at baseline and ensuring the validity of subsequent group comparisons. Before proceeding to the main analyses, the normality assumption was tested using the Shapiro–Wilk statistic. All score distributions were found to be approximately normal, with *p*-values exceeding the 0.05 criterion for the EG pre-test (0.843), CG pre-test (0.289), EG post-test (0.673), and CG post-test (0.391). Although the 0–100 scale could potentially introduce ceiling effects, supplementary non-parametric analyses (Mann–Whitney U tests) yielded results consistent with the parametric tests, thereby confirming the robustness of the findings.

**Figure 1:**
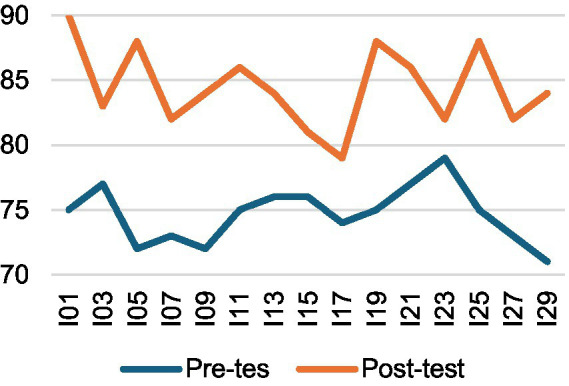
Interpreting performance changes—EG.

**Figure 2:**
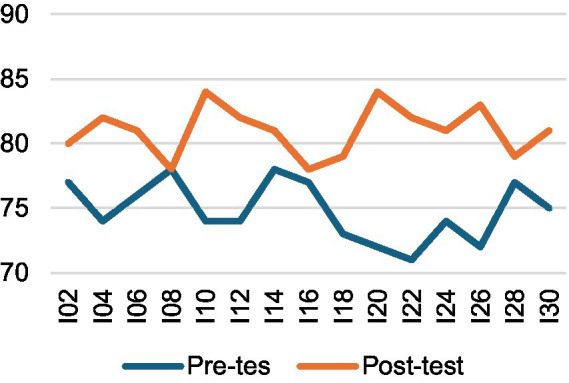
Interpreting performance changes—CG.

**Figure 3:**
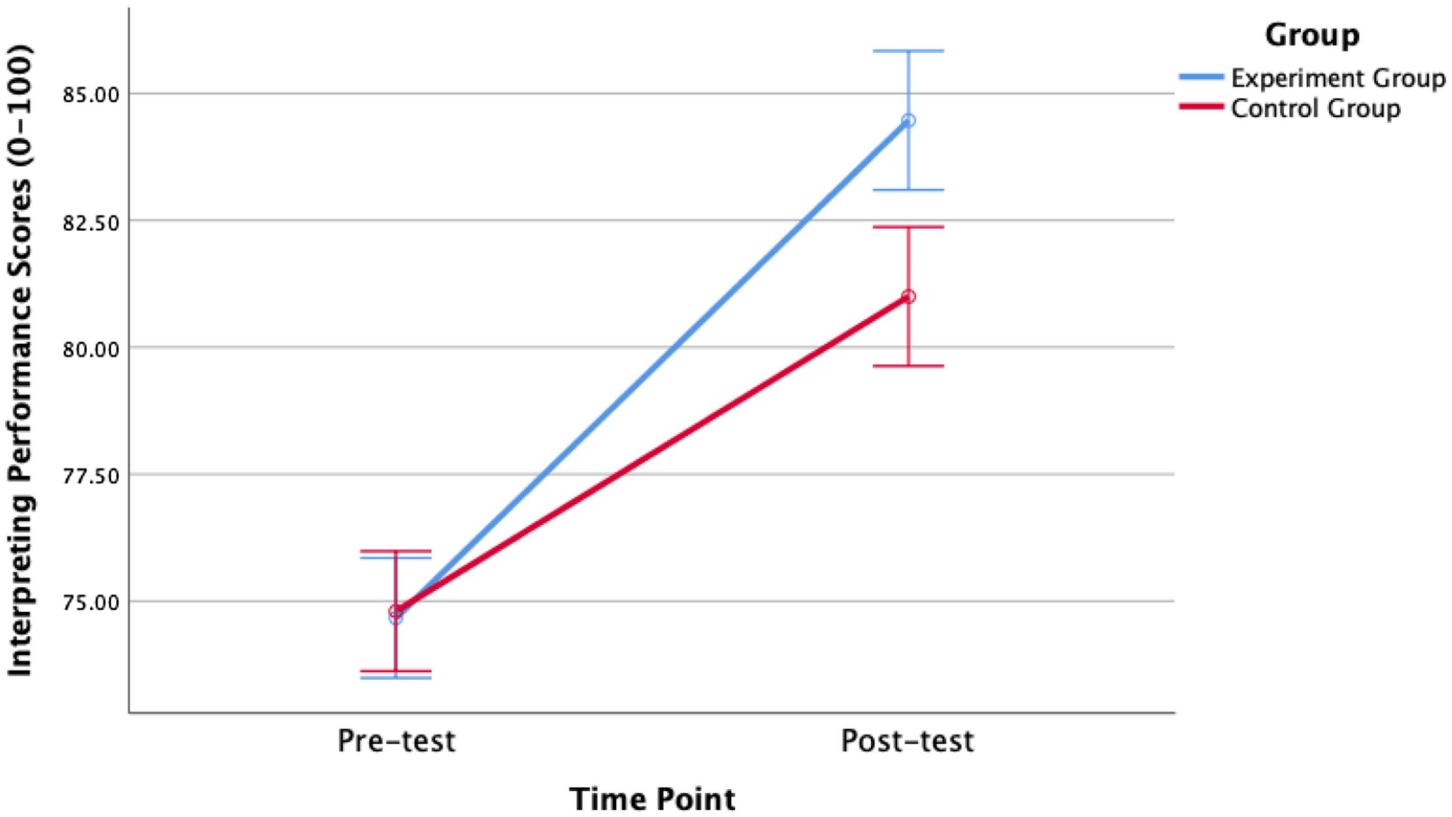
Changes in interpreting performance scores between groups.

A two-way mixed-design ANOVA (2 × 2) was then conducted, with time (pre-test vs. post-test) as a within-subjects factor and group (experimental vs. control) as a between-subjects factor. The results revealed a significant main effect of time, *F*(1, 28) = 127.76, *p* < 0.001, η^2^ₚ = 0.82, indicating that participants’ interpreting performance improved substantially from pre-test (*M* = 74.73, SD = 2.20) to post-test (*M* = 82.73, SD = 3.10) across both groups. As illustrated in Figures 1, 2, both groups showed performance gains over time, although the improvement was more pronounced in the experimental group. A significant time × group interaction, *F*(1, 28) = 9.97, *p* = 0.004, η^2^ₚ = 0.26, further confirmed that the intervention had a greater positive effect on the experimental group. According to Cohen’s (2013) benchmarks, these effect sizes are large, with time explaining 82% of the variance and the interaction accounting for 26.2%. Follow-up analyses using independent-samples *t*-tests on gain scores (post–pre) showed that the experimental group [*M* = 9.80, SD = 3.91, 95% CI (7.63, 11.97)] achieved significantly larger improvements than the control group [*M* = 6.20, SD = 3.84, 95% CI (4.07, 8.33)], *t*(28) = 2.54, *p* = 0.017, Hedges’ *g* = 0.90 [95% CI (0.14, 1.65)]. These findings indicate that although both groups demonstrated learning progress, the peer assessment intervention produced notably greater gains for the experimental group. The overall performance trend is depicted in Figure 3.

**Figure 4:**
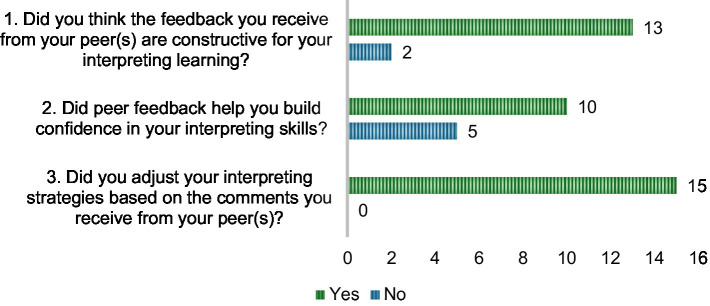
Student perceived impact of peer assessment on their interpreting performance.

The survey results further supported the RQ1. As shown in Figure 4, a large majority of students perceived peer assessment as beneficial to their interpreting performance. For Question 1 (Did you think the feedback you receive from your peer(s) are constructive for your interpreting learning?), 13 out of 15 students (86.7%) agreed that the feedback they received from their peers was constructive for their interpreting learning, while only 2 students (13.3%) responded negatively. In Question 2 (Did peer feedback help you build confidence in your interpreting skills?), 10 students (66.7%) reported that peer feedback helped build their confidence in interpreting, although 5 (33.3%) did not agree on this view yet observe improvement in their interpreting performance. Notably, in Question 3 (Did you adjust your interpreting strategies based on the comments you receive from your peer(s)?), all 15 students (100%) indicated that they adjusted their interpreting strategies based on the comments received from peers. These results suggest that peer assessment played a key role in raising students’ awareness of their performance and encouraging actionable improvement, even if its impact on confidence varied among individuals.

#### 4.1.2 Qualitative results

Five key themes emerged regarding student interpreters’ perceptions of how peer assessment influenced their interpreting performance. Each theme highlighted specific benefits and advantages attributed to peer assessment. These qualitative insights align with and reinforce the quantitative findings, thereby providing triangulated evidence in response to RQ1.

##### 4.1.2.1 Theme 1: increased awareness of specific weaknesses

This theme revealed that peer assessment significantly enhanced student interpreters’ awareness of specific weaknesses in their interpreting performance. Many students reported that feedback from peers helped them notice recurring issues they had previously overlooked, such as literal translation, poor pacing, or missing critical content like numbers or names. For example, one participant shared that he “*often missed translating numbers… especially when they were mentioned quickly (Student I03)*” and began to practice number-heavy speeches to improve accuracy. Moreover, one participant (Student I11) reflected that her peer told her interpreting “*lacked structure,*” prompting her to use sequencing words like “*first*” “*then*” and “*finally*” to make her output more organized. Several participant (Student I03, I09 and I13) emphasized how peer feedback exposed “*awkward sentence structures*” and helped them shift from word-for-word translation to more natural paraphrasing. These insights illustrate that peer comments served as a diagnostic tool and also as a catalyst for strategic adjustments. The immediacy and relatability of peer feedback allowed students to recognize weaknesses in a non-threatening environment and take timely action to refine their interpreting skills.

##### 4.1.2.2 Theme 2: improved delivery and fluency

The theme 2 revealed that peer assessment played a meaningful role in helping student interpreters enhance their delivery and fluency during interpreting tasks. Many students reported that peer feedback made them more aware of how their tone, pacing, and speaking style affected the clarity and professionalism of their performance. For instance, one participant mentioned that he was told his “*intonation was flat and boring (Student I09)*,” which led him to *“practice with emotion and emphasis”* to sound more confident and natural. Another participant also shared that her peer pointed out their habit of using filler words, so she began *“pausing silently instead of filling the gaps (Student I15),”* which significantly improved the smoothness of her speech. Some students also noted that peer comments prompted them to speak louder and with clearer articulation, as one participant recalled being told her voice was *“too soft and unclear (Student I19)”* motivating her to monitor her volume and intonation more closely. These findings thus suggest that peer assessment encouraged interpreters to shift their focus beyond content accuracy and to consider the listener’s experience, resulting in more polished and effective delivery.

##### 4.1.2.3 Theme 3: strategy adjustment and technique refinement

This theme found that peer assessment benefits student interpreters by encouraging to adjust their interpreting strategies and refine techniques based on received assessment. Participants frequently described making specific, targeted changes in response to the feedback they received. For example, one participant explained that after being told her “*note-taking lacked structure (Student I15)*,” she modified her learning method by incorporating more symbols and arrows to improve coherence. Another participant reflected that a peer suggested “*finishing thoughts instead of cutting off mid-sentence (Student I21),*” which prompted her to practice more complete and logically connected outputs. Another participant also shared that she “*used to translate everything literally (Student I23)*” but after peer feedback, began experimenting with paraphrasing to sound more natural. Students also reported revising their pacing, time management, and even preparation habits in response to repeated peer observations. These perspectives suggest that peer feedback provided a practical framework for self-monitoring, encouraging students to shift from passive reception to active strategy implementation, enabling them to achieve polished and effective interpreting performances.

##### 4.1.2.4 Theme 4: confidence and motivation boost

This theme finds that peer assessment contributed to skill development and at the same time, played a crucial role in boosting students’ confidence and motivation during interpreting training. Many participants expressed that receiving feedback from peers helped reduce their anxiety and encouraged them to keep improving. For example, one participant noted that hearing positive comments made her *“feel more confident and less nervous in class (Student I05)”* while another participant said peer feedback felt *“like advice from a teammate, not criticism (Student I15),”* making it easier to accept and apply suggestions. Several participants also mentioned feeling seen and supported, with one claiming that peer feedback *“gave me more confidence because it came more frequently and felt more personal (Student I17).”* These experiences suggest that the non-judgmental and collaborative nature of peer assessment created an emotionally safe learning environment, thus students were more willing to take risks, stay engaged, and persist in refining their interpreting skills under this attachment. As a result, peer interactions acted as performance evaluators and also as motivators that sustained students’ enthusiasm and commitment throughout the interpreting training.

##### 4.1.2.5 Theme 5: peer feedback as a practical and accessible tool

The last theme further points out that students viewed peer assessment as informative, practical and accessible tool that supported sustained learning. Unlike teacher feedback, which some participants found less frequent or harder to act on, peer feedback was described by one participant as *“more regular and easier to apply (Student I09).”* Several students emphasized the immediacy and relatability of comments from their group members, with one participant noting that peer feedback was *“more specific to what we had just practiced (Student I13),”* making it timely and directly relevant. In addition, one participant reflected that peer suggestions were *“relevant and achievable (Student I29),”* particularly because they came from individuals at a similar skill level who understood the same challenges. This made the feedback feel more actionable and less intimidating. Furthermore, students appreciated the two-way nature of peer evaluation, where both giving and receiving feedback enhanced their awareness and engagement. Overall, their responses suggest that peer assessment functioned as a learning aid and also as a sustainable and accessible alternative to instructor-led evaluation, offering consistent and meaningful input throughout the interpreting training.

### 4.2 Relating to RQ2

#### 4.2.1 Quantitative results

**Figure 5:**
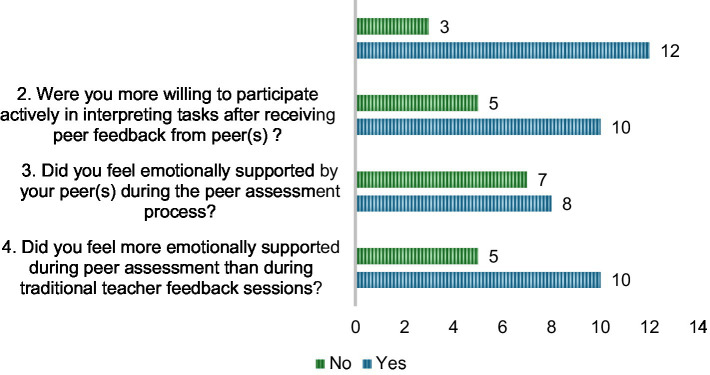
Students’ perceived impact of peer assessment on learning engagement.

With regard to RQ2 (Is peer assessment associated with improved learning engagement among student interpreters?), the survey results illustrated in Figure 5 indicate that peer assessment was generally perceived to enhance learning engagement among student interpreters. A majority of participants reported positive emotional and behavioral effects: 12 out of 15 students (80%) agreed that having a consistent partnership during peer assessment made them feel more comfortable taking risks in interpreting tasks, and 10 students (66.7%) felt more emotionally supported in peer assessment compared to previous traditional teacher feedback sessions. Similarly, 10 students (66.7%) indicated that receiving feedback from peers increased their willingness to participate actively in interpreting tasks. Emotional support from peers during the peer assessment process was acknowledged by just over half of the students (8 students, 53.3%), suggesting that while the practice fostered engagement for many, the degree of perceived emotional support varied among individuals.

For those who did not perceive such benefits, it might be due to lower degrees of attachment between group members, which could often contribute to personality clashes between peers or concerns about the quality and accuracy of peer feedback. As Han and Zhao (2020) have shown that the accuracy of peer ratings can be influenced by factors such as rater experience, the directionality of interpreting, and the inherent difficulty of assessing certain quality dimensions (e.g., fluency, expression). In cases where peer raters were less experienced or feedback was perceived as unhelpful, the engagement-enhancing effect of peer assessment may have been diminished. Overall, these findings suggest that while peer assessment is often associated with increased comfort, emotional support, and participation, its effectiveness depends on degrees of peer attachment and the reliability of peer feedback.

#### 4.2.2 Qualitative results

Four themes were identified from thematic analysis. Each theme uncovers the unique perspective of influence of peer assessment on student interpreters’ engagement in the class. These qualitative results further answer RQ2.

##### 4.2.2.1 Theme 1: peer relationships foster emotional safety and motivation

This theme finds that peer assessment fostered emotional safety and motivation among student interpreters, which in turn enhanced their learning engagement. Many students expressed that working with close peers created a sense of trust and emotional comfort that allowed them to take more risks and be more open to learning. For example, one participant described feeling “*more relaxed when receiving peer feedback (Student I03),*” explaining it felt like “*a conversation between friends*” rather than formal evaluation. Another participant reflected that “*we trusted each other, I wasn’t afraid to make mistakes (Student I07)*” and this supportive relationship encouraged him to try new strategies in class. Surprisinly, some participants (*Student I17 and I19*) contrasted this with teacher feedback, which often felt “*more critical*” or “*too formal and distant*” while peer input was “*simpler, more relatable*” and emotionally encouraging. This safe environment thus lowered anxiety and strengthened motivation, as students were more willing to participate and persist. Participant also stated, “*having a friend to lean on made the experience more positive (Student I23)*.” Overall, these findings suggest that peer relationships cultivated through assessment practices were instrumental in building an engaging and emotionally secure classroom climate.

##### 4.2.2.2 Theme 2: increased engagement through social accountability and partnership

This theme suggests that peer assessment enhanced student engagement by fostering a sense of social accountability and partnership. Many participants reported that knowing their peers were relying on them for feedback, and vice versa, made them more attentive and responsible during interpreting tasks. For instance, one participant shared that he “*did not want to let my partner down (Student I03)*,” which motivated him to stay focused and well-prepared for class. Another participant also emphasized that the consistent peer interaction created “*a team-like environment*” where “*everyone cared about each other’s progress (Student I09)*.” Several students noted that peer work created a feeling of shared responsibility, as they were not only interpreters but also evaluators, which made them more engaged throughout the assessment activity. As one participant put it, “*I had to listen carefully to give good feedback, so I stayed alert even when it wasn’t my turn (Student I23).*” Another participant added that she felt “*more invested (Student I19)*” in the learning process because peer assessment was not just about personal performance but about helping the group improve together. These responses collectively highlight how peer partnerships elevated classroom participation by establishing mutual expectations, encouraging accountability, and deepening the students’ emotional and cognitive investment in the learning experience.

##### 4.2.2.3 Theme 3: enhanced focus and active participation

This theme demonstrates that peer assessment encouraged greater focus and active participation during interpreting tasks. Most participants reported being more mentally engaged because they were not only responsible for interpreting tasks but also for evaluating their peers. For example, one participant explained that she “*could not just sit back and listen*” because she had to “*pay close attention to give fair and useful feedback (Student I01).*” Another respondent noted that the dual role of being both interpreter and assessor made the activity “*more interactive and less passive (Student I09)*” than traditional teacher-led sessions. In addition, one participant said that peer assessment kept her on task for the entire class period, as she were “*constantly thinking about how to improve both myself and my partner (Student I19)*.” Moreover, one participant highlighted that this structure encouraged her to stay tentatively at class even when not performing, since she was “*learning by observing and listening to classmates carefully (Student I23).*” Another participant also added that “*the need to give feedback made me more aware of what makes a good interpretation (Student I29)*,” which deepened her learning engagement. These comments suggest that the interactive, collaborative nature of peer assessment fostered a more cognitively stimulating learning environment that encouraged sustained focus and meaningful participation.

##### 4.2.2.4 Theme 4: increased confidence and sustained engagement through encouragement

This theme shows that peer encouragement played a significant role in boosting students’ confidence and sustaining their engagement throughout the interpreting training. Many respondents described how peer feedback was corrective and uplifting, helping them feel seen and valued. For example, one participant shared that her peer’s praise made her “*realize I was improving (Student I21)*,” which “*motivated me to keep going even when I felt stuck.*” Another respondent noted that “*positive feedback from classmates gave me more courage to speak up even during challenging tasks (Student I15).*” Moreover, several respondents emphasized that peer support helped reduce self-doubt. For instance, one respondent shared that “*my classmate’s encouragement made me feel more capable, which helped me participate more actively in later sessions (Student I19).*” In addition, another respondent mentioned that hearing his peer say “*I liked how you handled the numbers (Student I27)*” gave him a confidence boost that lasted after that session. Some other participants (*Student I13, I17 and I29*) said that regular peer feedback helped them *“stay motivated”* because they could *“see progress over time*,” reinforcing their long-term commitment to the class. These findings highlight that beyond its academic function, peer assessment fostered emotional resilience and long-term motivation by creating a secure supportive and affirming interpersonal attachment.

### 4.3 Relating to RQ3

#### 4.3.1 Quantitative results

**Figure 6:**
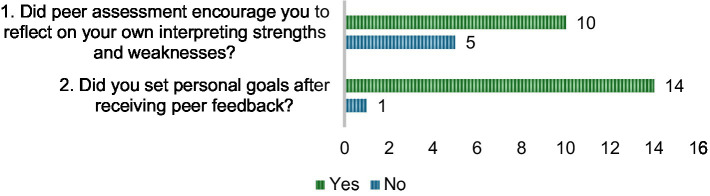
Student perceived impact of peer assessment on self-regulated learning.

Regarding RQ 3 (Is peer assessment associated with improved SRL among student interpreters?), as shown in Figure 6, peer assessment was generally perceived to support the development of SRL among student interpreters. Fourteen out of fifteen students (93.3%) reported that they set personal goals after receiving peer feedback, suggesting that the process effectively encouraged learners to take proactive steps in directing their own progress. In addition, 10 students (66.7%) stated that peer assessment prompted them to reflect on their interpreting strengths and weaknesses, while 5 students (33.3%) did not perceive such an effect. This difference suggests that while most students benefited from the reflective opportunities provided by peer assessment, a significant minority did not fully engage in self-evaluation. From the perspective of Attachment Theory, students who felt secure and supported within their peer relationships may have been more willing to critically examine their own performance and set challenging goals, seeing feedback as constructive rather than threatening. In contrast, those with less secure peer connections or lower trust in their partners may have been less inclined to internalize feedback or engage deeply in reflection, focusing instead on task completion without self-assessment. This variation implies the role of perceived relational security in shaping the extent to which peer assessment fosters SRL in interpreter training.

#### 4.3.2 Qualitative results

Qualitative results further convince the impact of peer assessment on student interpreters’ SRL. Thematic analysis yield four themes, with student interpreters perceive peer assessment as effective learning strategies to improve their SRL in different perspectives such as reflective thinking, goal-setting, self-tailored practicing and so on.

##### 4.3.2.1 Theme 1: development of reflective thinking and self-awareness

This theme indicates that peer assessment significantly contributed to the development of student interpreters’ reflective thinking and self-awareness. Many participants reported that the process of both giving and receiving feedback helped them identify patterns in their performance and become more mindful of their strengths and weaknesses. For example, one participant noted that he started “*thinking more carefully about what went well and what needed improvement (Student I03)*” after each round of peer assessment, which fostered a habit of regular self-reflection. Another respondent shared that she “*used the rubric not just for giving feedback but also for checking my own performance (Student I21),*” showing how peer assessment served as a framework for self-evaluation. Moreover, participants also began comparing their own work to that of their peers, with one participant commenting that “*seeing how my peers performed… made me realize it wasn’t just me struggling (Student I15).*” This sense of shared learning thus helped normalize mistakes and enhanced critical thinking. Additionally, several participants mentioned that peer assessment pushed them toward greater independence, with one participant commenting, “*I do not need someone else to always tell me what’s wrong, because I can now catch some of my own errors (Student I23)*.” Overall, these responses suggest that peer assessment supported language and interpreting skill development and at the same time nurtured a deeper, self-directed learning approach.

##### 4.3.2.2 Theme 2: goal setting and strategic learning planning

This theme reveals that peer assessment encouraged students to set personal learning goals and engage in strategic planning to guide their interpreting improvement. Several participants described how repeated peer feedback helped them identify specific problem areas and take intentional steps to address them. For instance, one participant noted that “*I wrote down what my peers said and made a plan to work on one issue each week (Student I11)*,” showing how feedback became a tool for short-term goal setting. Similarly, one respondent shared that she “*started planning my practice more purposefully (Student I17)*” after realizing from feedback that she needed to improve her pacing. In addition, some respondents described that the desire to avoid repeating the same mistakes in future assessments motivated them to set weekly learning targets. For example, one participant mentioned, “*My peer said I lacked confidence in delivery, so I practiced in front of a mirror every day before class (Student I19)*.” Moreover, one participant also said he “*focus on doing business report in The Economist (Student I07)*” after being reminded of his misuse of business terminologies. These reflections suggests that peer assessment helped students become more aware of their weaknesses and also empowered them to take proactive and structured steps toward improvement.

##### 4.3.2.3 Theme 3: tailored and targeted practice habits

This theme shows that peer assessment motivated students to develop tailored and targeted practice habits that addressed their individual interpreting challenges. Many participants described how specific peer comments prompted them to create individualized practice routines. For example, one respondent explained that after receiving feedback on frequent hesitation, she began “*timing myself when interpreting to stay within the time limit (Student I17).*” Another respondent also reported that his peer noticed issues with number accuracy, so he “*made a habit of practicing number-heavy talks every day (Student I03)*” to strengthen his interpreting performance. Additionally, several respondents said that they customized their home practice to align with peer observations, such as “*shadowing short speeches with fast speakers to improve fluency (Student I25)*,” or “*practiced interpreting speeches with many figures and trained myself to write down numbers faster (Student I23)*” after struggling to interpret figures. Furthermore, one respondent recalled how she “*recorded my interpreting and compared it with the feedback (Student I15)*,” turning peer input into measurable targets for improvement. Another respondent also began “*reviewing grammar rules and doing exercises (Student I23)*” after being reminded of frequent tense errors. These diverse responses illustrate that peer assessment encouraged students to take ownership of their learning by designing practice tasks that directly targeted their weak points, making their preparation more focused, meaningful, and effective.

##### 4.3.2.4 Theme 4: use of peer feedback as a learning framework

The last theme indicates that many students began to internalize peer feedback as an integral part of their learning process, using it as a framework to guide both self-reflection and future improvement. Rather than viewing feedback as a one-time comment, students described using it to shape their ongoing learning routines. One participant explained that he “*built a checklist based on peer suggestions and used it every time I practiced (Student I09).*” Another respondent noted that she “*reflected on my peer’s feedback even after class to adjust how I interpreted the next session (Student I25)*,” showing that feedback became a reference point for sustained improvement. Several respondents also shared that giving peer feedback helped them become more aware of learning standards, with one respondent stating, “*evaluating others helped me apply the same standards to myself (Student I21)*.” Similarly, others began incorporating the feedback rubric into their own learning, such as one respondent claimed that she “*used the same criteria to self-assess after each recording (Student I29).*” Additionally, one respondent also highlighted that she “*set clearer goals when my peers pointed out areas to improve such as constant pause and hesitation fillers (Student I11),*” highlighting how feedback shaped the structure of her learning process. Altogether, these responses suggest that peer assessment enhanced interpreting performance and provided students with a self-directed, consistent, and reflective learning framework that extended beyond classroom practice.

## 5 Discussion

### 5.1 The impact of peer assessment on student interpreters’ interpreting performance, engagement, and self-regulated learning

This study aimed to examine the effects of peer assessment on student interpreters’ performance, learning engagement, and SRL. A quasi-experimental design compared an experimental group, which engaged in peer assessment, with a control group, which received only teacher feedback. Both groups improved in interpreting performance, but the peer assessment group achieved greater interpreting performance. Survey findings further triangulated the findings that most students perceived peer assessment as beneficial for reflection, goal-setting, and participation, while a smaller proportion did not report such benefits. These mixed responses highlight that although peer assessment can yield substantial pedagogical advantages, its impact is not universal, suggesting that degrees of peer attachment, individual learner differences and feedback conditions may influence outcomes.

Regarding RQ1 (association between peer assessment and interpreting performance), a greater improvement in interpreting performance in the experimental group than control group are consistent with research showing that dialogic, structured peer feedback enhances learning outcomes by engaging students as active evaluators (Cui et al., 2021; Er et al., 2020). Additionally, students who benefited most claimed observing peers’ work, receiving diverse perspectives, and reflecting with rubrics as critical factors, which aligns with the findings that peer input improved both competence and confidence (Ebadijalal and Yousofi, 2021; Li and Ke, 2022; Wang and Han, 2013). However, 33.3% of students in our study did not attribute performance improvement to peer assessment, often due to vague or inconsistent comments. As noted by Cao et al. (2022) and Jongsma et al. (2024), the effectiveness of peer feedback depends heavily on quality, trust, and psychological safety. Therefore, without explicit training or a dialogic framework, some students may not fully integrate feedback into performance improvement.

In relation to RQ2 (association between peer assessment and learning engagement), most students reported increased willingness to participate (66.7%) and greater risk-taking in performance (80.0%), aligning with Luan et al. (2020) and Liu et al. (2023) who highlight peer support and interaction as drivers of engagement. Nevertheless, 46.7% of participants did not feel emotionally supported, suggesting that for some, the peer assessment context lacked the perceived safety needed to encourage openness, which was also noted by Xu and Zhang (2025) in their sociocultural analysis of affective engagement. This disengagement may stem from lower degree of peer attachment, personality mismatches, competitive dynamics, or insufficient feedback dialogue, as described in Jongsma et al. (2024). Generally, these findings reinforce that engagement benefits are maximized when peer feedback is embedded in a collaborative, supportive, and secure environment that builds trust among peers.

For RQ3 (association between peer assessment and SRL), the present study finds that the majority of respondents reflected on strengths and weaknesses (66.7%) and set personal goals (93.3%) after receiving peer feedback, indicating that peer assessment can stimulate metacognitive processes crucial for SRL (Kumar et al., 2023; Ortega-Ruipérez and Correa-Gorospe, 2024; Theobald and Bellhäuser, 2022). This is also consistent with Rasheed et al. (2021) and Tran and Qing (2024), who found that structured peer activities promote monitoring, goal-setting, and self-correction. However, a small group did not engage in such reflection or goal-setting, possibly due to low confidence in the feedback source or uncertainty about how to act on it due to highly demanding interpreting tasks (Öztürk et al., 2025). This suggests that while peer assessment promotes SRL, it requires clear rubrics, scaffolding, and possibly technology-supported processes to ensure all learners can translate feedback into concrete strategies.

In conclusion, our findings confirm that peer assessment can positively influence interpreting performance, engagement, and SRL, but these benefits are contingent on the quality of feedback, the degree of emotional safety, and learners’ readiness to act on input. These findings highlight the potential of peer assessment to foster both cognitive and affective dimensions of learning. However, the findings also point out cautions that uneven benefits are common without systematic curriculum design and harmonious interpersonal relationships. To optimize interpreting training outcomes, interpreter trainers should integrate peer assessment with technology-assisted training, build supportive peer relationships, and provide structured opportunities to apply feedback.

### 5.2 Theoretical implications

The results of the present study map cleanly onto core propositions of Attachment Theory. Most students reported greater comfort taking risks (80%), higher willingness to participate (66.7%), strategy use (100% adjusted strategies), and SRL behaviors (93.3% set goals; 66.7% reflected) under peer assessment, alongside significant performance gains in the experimental group. This pattern is consistent with the “from secure base to exploration” mechanism (Bretherton, 1985). When interactions feel safe, learners are more willing to try, revise, and persist. It also aligns with models of feedback acceptance that hinge on psychological safety. Constructive, frequent, future-focused input is more likely to be taken up when relationships feel secure (London et al., 2023). However, the minority who did not feel emotionally supported (46.7%) or did not report reflection (33.3%) are partially associated with lower peer feedback acceptance, greater threat sensitivity, and dampening WTC and engage (Amirian et al., 2022). Moreover, the strong goal-setting and self-tailored practice echo attachment-informed accounts of how secure relational contexts scaffold learner autonomy in language learning (Toffoli, 2015).

Theoretically, the study extends Attachment Theory into adult interpreter training by specifying a peer-level mechanism in which high-quality peer attachment fosters psychological safety, which in turn promotes feedback uptake, encourages the development of SRL routines, and ultimately enhances interpreting performance (Jin et al., 2022). It also surfaces boundary conditions. First, mismatches in attachment-related interaction styles may blunt feedback acceptance (London et al., 2023), helping to explain mixed engagement and support ratings in our sample. Secondly, affect is not incidental. Where safety is weaker, anxiety likely suppresses risk-taking and participation (Amirian et al., 2022), limiting the SRL dividends of peer work. By positioning autonomy-relevant SRL behaviors (goal setting, targeted practice) as outcomes of safe peer feedback rather than only individual traits, our findings enrich attachment-informed accounts of learner autonomy (Toffoli, 2015) and broaden the theory’s application from teacher–student relationships (Ozturk, 2024) to student–student feedback dynamics in higher education.

### 5.3 Pedagogical implications

The findings of the present study highlight that peer assessment, when considerately designed and scaffolded, can significantly enhance interpreter trainees’ interpreting performance, learning engagement, and SRL. For future interpreting training, trainers should adopt a dialogic peer feedback approach that promotes active sense-making, co-construction of meaning, and follow-up action (Er et al., 2020; Jongsma et al., 2024; Massey and Brändli, 2016). Structured peer assessment activities should include preparatory guidance on providing balanced, constructive feedback, with explicit rubrics that address both micro-level linguistic accuracy and macro-level discourse organization (Ortega-Ruipérez and Correa-Gorospe, 2024; Su, 2019). Such scaffolding fosters higher cognitive engagement and strengthens affective engagement by creating a psychologically safe environment for criticism, as peer attachment quality can enhance psychological safety and, in turn, facilitate feedback uptake (London et al., 2023). Furthermore, embedding peer assessment into regular interpreting practice, rather than as isolated tasks, can cultivate sustainable SRL habits, as evidenced in studies showing peer feedback’s positive influence on motivation and strategic learning behaviors (Cui et al., 2021; Tran and Qing, 2024; Han, 2018a,b).

However, trainers should also be mindful of potential challenges, such as peer pressure, distrust, or overemphasis on surface-level feedback (Cao et al., 2022; Power and Tanner, 2023). To mitigate these risks, feedback sessions should balance anonymity with opportunities for constructive dialogue (Jongsma et al., 2024; Sha et al., 2022) and include reflective debriefs where trainees discuss how they have acted on feedback received. Trainers should also monitor the emotional dimension of feedback exchanges, as affective responses can strongly shape subsequent engagement and learning outcomes (Ajjawi et al., 2021; Xu and Zhang, 2025). Furthermore, integrating technology-mediated peer assessment, such as online platforms that allow structured, trackable exchanges, can enhance immediacy, traceability, and revision opportunities (Chang et al., 2023; Ebadijalal and Yousofi, 2021; Öztürk et al., 2025; Yang et al., 2025a,b; Zou et al., 2025). Last but not least, trainees should critically adopt peer assessment approach to ensure it is a well-orchestrated and characterized by clear criteria, psychological safety, structured dialogue, and iterative reflection so as to make it possible to internalize peer feedback, deepen SRL strategies, and achieve higher professional performance standards.

### 5.4 Conclusion, limitations and future research directions

This study explored how peer assessment can influence interpreter students’ interpreting performance, engagement, and SRL, underpinned by Attachment Theory. Using a quasi-experimental design with pre- and post-tests and a post-intervention survey, the research compared a peer-assessment group with a control group that received only teacher feedback. The findings showed that peer assessment enhanced interpreting performance and encouraged reflective learning, goal-setting, and greater willingness to engage in future self-improvement. While most participants reported benefits in emotional support and reflective practice, a small number expressed reservations, suggesting that the interpersonal and emotional aspects of peer feedback warrant careful consideration. Overall, the results point to peer assessment as a valuable tool for fostering both skill development and learner autonomy in interpreter training.

However, several limitations should be acknowledged. First, the quasi-experimental design relied on systematic sampling based on student ID rather than full randomization. Although baseline equivalence between the experimental and control groups was statistically confirmed, potential allocation bias cannot be completely ruled out, as student IDs may correlate with other unobserved factors such as class section or enrollment order. This limitation may affect the internal validity of the findings. Future research should therefore employ fully randomized controlled trials to minimize allocation bias and strengthen causal inference. In addition, the relatively small and homogeneous sample which consists entirely of L1 Chinese students with high English proficiency from a single institution, limits the generalizability of the results. Contextual factors such as course structure, interpreting task types, and instructional style may have influenced learning outcomes, bounding the transferability of the findings to other pedagogical settings. To enhance external validity, future studies should include larger and more diverse samples drawn from multiple institutions, covering different linguistic backgrounds, proficiency levels, and interpreter training contexts.

A second major limitation concerns differences in feedback exposure between groups. The experimental group received weekly peer feedback, whereas the control group obtained instructor feedback approximately every 2 weeks due to class time constraints. Although both groups accumulated comparable total feedback across the intervention period, variation in feedback frequency, immediacy, and interpersonal engagement may have influenced learning gains independently of feedback source. As a result, it is difficult to completely separate the effects of peer assessment from those of exposure to feedback itself. Future research should control for feedback exposure either by equalizing feedback frequency across conditions or by including feedback frequency as a covariate in statistical models such as ANCOVA. Sensitivity analyses could also be employed to assess whether the observed differences remain robust under matched exposure conditions. Furthermore, future studies may explore how feedback depth, timing, and learner receptivity contribute to the pedagogical effectiveness of peer and teacher feedback within interpreter training.

Third, although inter-rater reliability was established using intraclass correlation coefficients for overall interpreting scores, raters provided holistic rather than dimension-specific ratings based on the three criteria of Content, Form, and Delivery. This prevented sub-dimensional analyses that might have revealed which performance aspects such as accuracy, fluency, or delivery benefited most from the intervention. Future research should incorporate analytic rubrics with separate dimension ratings to enable a more diversified interpretation of where learning gains occur. Moreover, while this study reported mean scores and significance levels, it did not include delayed post-tests, which limits insight into the long-term retention of interpreting skills fostered through peer assessment. Incorporating delayed post-tests would help determine whether performance improvements persist over time. Additionally, the survey items assessing engagement, SRL, and emotional support were self-developed and analyzed at the item level without psychometric validation. Although conceptually aligned with the study’s theoretical framework, internal consistency such as Cronbach’s *α* was not established, and no standardized measures of attachment, psychological safety, or WTC were employed. Future research should therefore utilize validated multi-item instruments and assess internal consistency to strengthen the reliability and comparability of survey-based findings.

Finally, from a theoretical perspective, while the study was framed by Attachment Theory, attachment-relevant constructs such as peer trust, psychological safety, and WTC were not directly measured. Consequently, the mechanisms linking peer assessment to enhanced engagement and self-regulated learning remain inferential rather than empirically tested. Future research should incorporate validated measures such as the experiences in close relationships scale–short form (ECR-S) for attachment anxiety and avoidance, psychological safety scales, WTC instruments, and peer trust questionnaires to empirically examine these proposed pathways. Employing mediational or longitudinal designs would allow for testing whether peer assessment promotes engagement and performance through attachment-related mechanisms. Moreover, as the study was conducted entirely in a face-to-face classroom environment, it did not consider the potential influence of technology-mediated or AI-assisted peer assessment tools, which are becoming increasingly prevalent in interpreter training. Future studies could explore how digital feedback systems, AI-supported evaluation platforms, or online collaborative environments shape the emotional, cognitive, and social dimensions of peer assessment, providing richer insights into its evolving pedagogical role.

## Data Availability

The original contributions presented in the study are included in the article/Supplementary material, further inquiries can be directed to the corresponding authors.
