# Supplementary material for: Enhancing interpreting performance, engagement, and self-regulated learning through peer assessment: an attachment theory perspective
Source: Front Psychol. 2025 Dec 5;16:1604825. doi: 10.3389/fpsyg.2025.1604825 (PMC12714925; doi:10.3389/fpsyg.2025.1604825)
Supplement: Supplementary file 1 [file Supplementary_file_1.docx]

# **Appendix I Interpreting Assessment Rubric**

| **Marking Rubric for Consecutive Interpreting Performance (Lee, 2018)** | |
| --- | --- |
| Dimension | Peer / Instructor Assessment |
| **1. CONTENT (7 criteria)**  __ No opposite meanings  __ Accurate rendition of main ideas  __ No unjustified change in meaning  __ Logical cohesion  __ High level of completeness of information  __ Accurate rendition of numbers and names  __ No unjustified additions |  |
| **2. FORM (7 criteria)**  __ No incomplete sentences  __ Natural/idiomatic target-language expressions  __ Unambiguous and clear diction  __ Appropriate register and speech level  __ Little source-language interference  __ Correct terminology  __ Grammatical correctness |  |
| **3. DELIVERY (7 criteria)**  __ Fluency of delivery (general concept/impression)  __ No significant repairs or backtracking  __ Impression of confidence  __ Few fillers, hesitations and pauses  __ Lively intonation and stress  __ Finishing interpretation within the time limit  __ No slips of the tongue |  |
| **Total** | ( ) / 100 |

# **Appendix II Excerpt Transcript of Interpreting Training Materials**

| Scenario 1 | A Tourist Visiting in Dalian City, China | |
| --- | --- | --- |
| Setting | A foreign tourist (A) is visiting Dalian for the first time. He meets a local tour guide (B) who introduces the city’s attractions, culture, and cuisine. | |
| A | 欢迎您来到大连！大连是中国东北最美丽的海滨城市之一。您是刚到吗？ | |
| B | Yes, I came by high-speed train from Beijing. The journey was smooth and the sea view near Dalian was amazing! | |
| A | 大连以干净的环境、宜人的气候和欧式建筑而闻名。这里每年都会举办国际服装节和啤酒节。 | |
| B | I see. That sounds wonderful. Is the city big? | |
| A | 不算大，但非常现代、规划得很好。城市三面环海。 | |
| B | What are the must-see places in Dalian? | |
| A | 您一定不能错过星海广场，它是亚洲最大的城市广场。老虎滩海洋公园也很受欢迎。如果您喜欢自然风光，滨海路是个自驾或散步的好去处。 | |
| B | Sounds great! I’d love to take some photos there. | |
| A | 对了，您一定要尝尝这里的海鲜，尤其是扇贝、海参和烤鱿鱼。大连人特别喜欢吃新鲜的海味。 | |
| B | I can’t wait! Is there a night market where I can try local snacks? | |
| A | 有的，在青泥洼桥附近就有一个夜市，什么小吃都有。 | |
| B | Thank you for the wonderful introduction. Dalian is really impressive! | |
| A | 不客气！希望您在这里度过愉快的时光，带回美好的回忆。 | |
|  | | |
| Glossary | | |
| 1 | 滨海城市 | costal city |
| 2 | 高铁 | high-speed train |
| 3 | 国际服装节 | International Fashion Festival |
| 4 | 啤酒节 | Beer Festival |
| 5 | 星海广场 | Xinghai Square |
| 6 | 老虎滩海洋公园 | Laohutan Ocean Park |
| 7 | 滨海路 | Binghai Road |
| 8 | 扇贝 | scallop |
| 9 | 海参 | sea cucumber |
| 10 | 烤鱿鱼 | grilled squid |
| 11 | 青泥洼桥 | Qingniwa Bridge |
| 12 | 夜市 | night market |
